# Selected Essential and Toxic Chemical Elements in Hypothyroidism—A Literature Review (2001–2021)

**DOI:** 10.3390/ijms221810147

**Published:** 2021-09-20

**Authors:** Anna Błażewicz, Patrycja Wiśniewska, Katarzyna Skórzyńska-Dziduszko

**Affiliations:** 1 Department of Pathobiochemistry and Interdisciplinary Applications of Ion Chromatography, Faculty of Biomedicine, Medical University of Lublin, 1 Chodźki Street, 20-093 Lublin, Poland; patrycja.wisniewska@umlub.pl; 2 Department of Human Physiology, Faculty of Medicine, Medical University of Lublin, 11 Radziwiłłowska Street, 20-080 Lublin, Poland; katarzyna.skorzynska-dziduszko@umlub.pl

**Keywords:** chemical elements, hypothyroidism

## Abstract

Thyroid hormones are known for controlling metabolism of lipids, carbohydrates, proteins, minerals, and electrolytes and for regulating body temperature. Normal thyroid status depends on the chemical/elemental composition of body fluids and tissues, which changes depending on physiological state, lifestyle and environment. A deficiency or excess of certain essential chemical elements (selenium, zinc, copper, iron or fluorine) or exposure to toxic (cadmium or lead) or potentially toxic elements (manganese or chromium) interacts with thyroid hormone synthesis and may disturb thyroid homeostasis. In our review, accessible databases (Scopus, PubMed and Web of Science) were searched for articles from 2001–2021 on the influence of selected chemical elements on the development of hypothyroidism. Our review adopted some of the strengths of a systematic review. After non-eligible reports were rejected, 29 remaining articles were reviewed. The review found that disruption of the physiological levels of elements in the body adversely affects the functioning of cells and tissues, which can lead to the development of disease.

## 1. Introduction

The thyroid gland is one of the most important organs of the endocrine system. It is also part of the hypothalamic–pituitary–thyroid (HPT) axis and is regulated by a negative feedback loop [1]. The thyroid gland is responsible for the production and secretion of two thyroid hormones: 3,5,3′,5′ - tetraiodothyronine (thyroxine; T4) and 3,5,3′- triiodothyronine (T3). T4 is produced by the thyroid gland, whereas T3 is also produced by the thyroid, but primarily in extrathyroidal tissues through deiodination of T4 [2]. Thyroid hormones are well known for controlling metabolism of lipids, carbohydrates, proteins, minerals, and electrolytes and for regulating body temperature [3]. An inadequate concentration of thyroid hormones affects nearly every organ or system, with consequences for metabolic, gastrointestinal, cardiovascular and neurological function [4]. An abnormally low concentration of T3 is responsible for the biochemical and clinical symptoms of hypothyroidism [2]. The most common autoimmune disease and the main cause of hypothyroidism is Hashimoto thyroiditis [5]. Depending on the definition used, the prevalence of overt hypothyroidism is estimated to be from 0–2% to 5–3% in the general population of Europe, and from 0–3% to 3–7% in the USA [6]. However, the prevalence of undiagnosed hypothyroidism in Europe may be even higher, estimated at 4.11% for subclinical hypothyroidism, 0.65% for overt hypothyroidism, and 4.70% in total. Hypothyroidism occurs more frequently among women (about 4–10/1, female/male) and the elderly (≥65 years) [7,8].

Hypothyroidism is thyroid hormone deficiency; the thyroid gland does not produce a sufficient level of hormones to maintain a physiological level of thyroid hormone signaling in the peripheral tissues. There are three types of hypothyroidism: primary, secondary and tertiary. Primary hypothyroidism results from a low level of thyroid hormone due to destruction of the thyroid gland. This condition results in increased secretion and elevation of serum thyroid-stimulating hormone (TSH) levels. If the structure of the gland remains normal, dysfunction can be caused by decreased TSH secretion from the pituitary; this is called secondary hypothyroidism. In tertiary hypothyroidism, a decrease in thyroid hormone arises from inadequate secretion of thyrotropin-releasing hormone (TRH) from the hypothalamus. It is not always possible to differentiate between secondary and tertiary hypothyroidism, and they are often collectively referred to as central hypothyroidism. About 99% of hypothyroidism cases are primary hypothyroidism [9]. The most common cause of primary hypothyroidism in iodine-sufficient areas is Hashimoto’s thyroiditis (a chronic autoimmune disorder) [10]. In many countries, preventive programs have been introduced to reduce the incidence of diseases caused by iodine deficiency, mainly by enriching food with this element. However, while these measures have eliminated severe iodine deficiencies, they may lead to excessive iodine intake and an increase in the incidence of autoimmune thyroid disease [11]. Excessive iodine intake is linked to a higher prevalence of autoimmune thyroiditis, while in iodine-deficient areas its prevalence is lower [12]. Subclinical hypothyroidism (SCH) is diagnosed when levels of serum free thyroxine (T4) and triiodothyronine (T3) are within the normal reference laboratory range, but the thyroid-stimulating hormone (TSH) concentration is increased. The progression of SCH may lead to overt hypothyroidism [9]. The clinical hallmarks of hypothyroidism include slowing down of metabolic processes and increasing edema, as a result of deposition of fibronectin and hydrolyzed glycosaminoglycans in the hypodermic tissue, muscles, and other tissues [10]. Levothyroxine (LT4) monotherapy is the current standard for management of central and primary hypothyroidism [13]. 

Most important trace elements in hypothyroidism include iodine (I), selenium (Se), zinc (Zn), manganese (Mn), chromium (Cr), copper (Cu), iron (Fe), fluorine (F) and lead (Pb). Iodine is necessary for the production of thyroid hormone [14]. Selenium is required for antioxidant function and for metabolism of thyroid hormones [15]. Zinc is involved in thyroid hormone synthesis and metabolism, conversion of T4 to T3, receptor activity, and production of carrier proteins [16]. Iron deficiency impairs thyroid metabolism. Adults with Fe deficiency have lower circulating concentrations of FT3 and FT4 and higher concentrations of TSH or thyrotropin than controls [17]. Manganese is a component of some enzymes and may affect thyroid hormone homeostasis [18]. High fluoride ingestion reduces T4 and T3 levels and leads to an abnormal increase in TSH levels [19].

Normal thyroid status depends on the chemical/elemental composition of body fluids and tissues, which changes depending on physiological state, lifestyle and environment. Certain levels of elements in tissues and body fluids are considered to be normal [20,21]. Deficiencies or excess amounts of these elements, disrupting their physiological levels in the body, adversely affect the functioning of the cells and tissues, which can lead to the development of disease. Another important consideration is the presence of toxic chemical elements in the body, which can compete with essential elements even at low exposure levels. Heavy metals tend to accumulate in the body [22]. This paper is a summary of current studies based on measurement of the concentrations of selected trace elements, i.e., Se, Zn, Mn, Cr, Fe, F, Cd and Pb, in patients with primary or secondary hypothyroidism. Current literature on this subject emphasizes and provides evidence that exposure to certain chemical elements (e.g., heavy metals), contributes to the etiopathogenesis of hypothyroidism and the autoimmune process. Despite the large number of studies conducted over many years [23,24,25,26,27,28,29,30,31,32,33,34,35,36,37,38,39,40,41,42,43,44,45,46,47,48,49,50,51,52], there are still no clear conclusions regarding correlations between the concentrations of chemical elements and the occurrence of hypothyroidism and the intensified autoimmune process associated with it. The latest research is revealing new data, so there is a need to assess the current state of knowledge in this area.

## 2. Methods

### 2.1. Search Strategy 

The review covers articles presenting reports of analysis of chemical elements in hypothyroidism was performed. The Scopus, PubMed and Web of Science electronic databases were searched. Data for the review were collected from January 2001 to May 2021. The following keywords were used in the search: “hypothyroidism” in conjunction with “trace elements”, “selenium”, “zinc”, “manganese”, “chromium”, “cadmium”, “copper”, “iron”, “fluorine” and “lead”.

### 2.2. Eligibility Criteria

To find the articles for the review, the following inclusion and exclusion criteria were applied (Table 1) [24].

### 2.3. Study Selection

**Figure 1:**
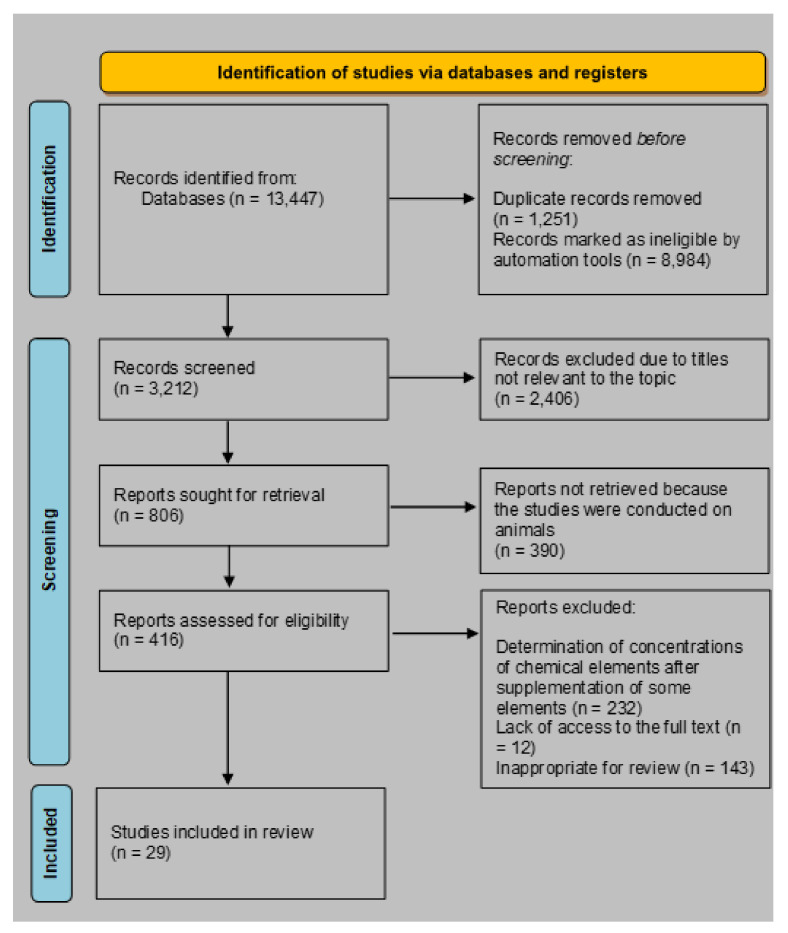
PRISMA (Preferred Reporting Items for Systematic Reviews and Meta Analyses) flowchart.

Our review adopted some of the strengths of a systematic review. The article selection is represented in a Preferred Reporting Items for Systematic Reviews and Meta Analyses (PRISMA 2020) flowchart (Figure 1).

## 3. Results and Discussion

The search identified 3212 references, which were screened in three steps—title, abstract, and entire text. A total of 29 publications were eligible for this review. Due to the high heterogeneity of published studies, the systematic review was difficult to prepare.

Typically, the contents of the chemical elements in relation to hypothyroidism were determined in serum [24,25,26,27,28,29,30,31,32,33,34,35,36,37,38,39,40,41,42,43,44,45], blood [25,46,47], plasma [48,49,50] or urine [42,46,47,51]. The chemical, electrochemical, optical spectrometry, X-ray spectrometry, mass spectrometry, and nuclear methods are commonly used for the analyses. Atomic Absorption Spectrometry (AAS) proved to be the most popular analytical technique used especially for Se, Zn, Mn, Cr, Cu, Fe, and Pb determinations [27,28,29,31,35,36,37,38,40,45,46,48,50]. Inductively Coupled Plasma Mass Spectrometry (ICP MS) was applied for Mn, Cu, Zn, Se, Pb, Cd, Cr, and F [30,34,39,43,47], whereas Neutron Activation Analysis (NAA) for Fe, Cu, Zn, Se, respectively [25,52]. Methodologies applying Total Reflection X-ray Fluorescence (TXRF) for Se, Cu, Zn [24,26,34] and potentiometric measurements by the use of ion selective electrodes for F [41,42,51] were described. Additionally, coupled analytical techniques like High-Performance Liquid Chromatography (HPLC)—Inductively Coupled Plasma Triple Quadrupole Mass Spectrometry (QQQ-ICP) was used to determine quantitatively Cr, Mn, Fe, Cu, Zn, Se, Cd, Pb [44]. Less common were colorimetric assays for Fe, Zn, and Cu [32,33].

As it was mentioned above, our review does not include results of iodine determinations because it is the most studied element in the context of thyroid pathology including hypothyroidism, and both the excessive states and deficiency of iodine have been described and can be found elsewhere [53]. However, an immunological context, in which iodine can mediate thyroiditis by at least two mechanisms is worth emphasizing. First, iodine can modify the thyroglobulin (Tg) molecule and lead to the formation of iodinated neoantigenic determinants to which immune tolerance has not been adjusted, or change the processing of Tg to facilitate generation of pathogenic but cryptic Tg determinants that may not contain iodine. Alternatively, iodine may cause apoptotic/necrotic effects on thyrocytes. In that case an increased level of thyroid antigens, which may activate autoreactive T cells in situ or in thyroid-draining lymph nodes, will be released. These mechanisms may act independently or in synergy, depending on the individual [54].

### 3.1. Selenium

The mutual interactions between dietary iodine and selenium and their influence on the activities of the selenoenzymes and the thyroid hormones thyroxine (T4) and triiodothyronine (T3) are well-studied [55]. Limited or inadequate supply of iodine and selenium may affect metabolism of both elements [56]. Se concentration is higher in the thyroid gland (0.2-2 μg/g) than in any other tissues. It is an integral component of selenoproteins in their active site [15]. Selenoproteins comprise three families of enzymes: glutathione peroxidases (GPXs), thioredoxin reductases (TRs) and deiodinases. Glutathione peroxidases have an important function in the reduction of peroxides and protect cells against the harmful activity of oxygen free radicals. Thioredoxin reductases are critical in antioxidant processes but are also integrated into gene expression and regulation of some transcription factors [57]. Deiodinases include three types: type 1, 2 and 3 iodothyronine deiodinases. Their common function is mediating the activation and inactivation of thyroid hormone, while the specific differences between them are the result of their catalytic properties and distribution in tissue [58]. The recommended intake of selenium for normal selenoprotein function is estimated at 60 to 70 μg per day.

Selenium deficiency may affect thyroid function as a result of decreased activity of glutathione peroxidases, which is associated with the intensity of oxidative damage, or of deiodinases, which can lead to deterioration of thyroid activity. The study by Khorasani et al. [48] showed that selenium deficiency affects levels of circulating thyroid hormones. Se status was measured with the participation of 23 hypothyroid children. The plasma selenium level (mean ± SD) was 98.79 ± 13.63 μg/L, with 26.08% children having low selenium levels (selenium deficiency cut-off <90.0 μg/L). There were significant positive correlations between selenium level and TSH, the children’s weight and their age. Selenium concentration was significantly inversely correlated with T4 level. The authors concluded that in pediatric hypothyroid patients, the required levothyroxine dose may depend on the level of selenium deficiency. Establishing this relationship requires greater attention to the selenium level in children with hypothyroidism, and selenium supplementation may be helpful in treating the disease.

An interesting cross-sectional study of 323 patients assessed the status of a variety of trace elements, including Se, in thyroid patients [48]. The subjects were categorized into two groups depending on the type of diagnosis: hypothyroidism (*n* = 61) and autoimmune thyroiditis (*n* = 107). There were significant differences in serum concentrations of Se between patients and controls. Serum Se concentrations were relatively low as compared to the group of healthy subjects. There was no relationship between the type of thyroid disease and a pronounced selenium deficiency.

Stojsavljević et al. [46] published a paper in which the concentrations of trace elements, including selenium, were examined. Serum levels of elements were measured in 93 patients (23 with hypothyroidism and 70 healthy subjects). The concentrations of selenium were higher (*p* < 0.0001) in the group of respondents suffering from hypothyroidism. The results indicated clear differences in element profiles between hypothyroidism and healthy subjects, indicating that this could be used as a unique profile of hypothyroidism.

Guo et al. [30] published a paper describing a prospective birth cohort study which examined the association between low selenium concentration and hypothyroidism during pregnancy, as well as the association between maternal low thyroid function and the birth size of infants. The subjects of the study were 1931 pregnant women from Shanghai in gestational week 28-36. The authors measured maternal serum selenium concentration, TSH levels, and the birth weights and lengths of newborns. Maternal serum Se levels were 136.9 ± 47.9 μg/L. Infant birth weights and lengths were 3.4 ± 0.4 kg and 49.9 ±1.9 cm. A significant association was observed between maternal serum Se levels (<103.7 μg/L) and TSH levels. Each unit increase in Se levels was correlated with a decrease of 0.014 μIU/mL in TSH levels; however, this correlation did not exist when serum levels were ≥103.7 μg/L. A significant inverse association was found between maternal TSH levels and infant birth weight. The results indicate that a low Se concentration in pregnant women may be associated with hypothyroidism, which may be linked to lower birth weight in infants.

Interestingly, according to the study by Maouche et al. [50], abnormal levels of selenium could be used as an indicator of thyroid oxidative damage in patients predisposed to dysthyroidism. The aim of this study was to investigate the associations between trace elements, including selenium, and oxidative stress markers (total antioxidant status (TAS), glutathione peroxidase (GPx)*,* and superoxide dismutase (SOD)) as well as indices of thyroid hormone disorders (TSH, FT4, FT3, Anti-TPO-Ab) and insulin resistance syndrome (IRS). The participants were classified into two untreated groups: subclinical hypothyroidism (SCH) and overt hypothyroidism (OH). Se levels were significantly lower in OH and SCH groups compared to controls. The authors observed that the pathophysiology of dysthyroidism is linked to thyrotropic axis disturbance and also to antioxidant disturbances. A correlation was found between thyroid hormone disorders (especially the TSH variant) and the status of antioxidant trace elements. The results indicate that a low level of selenium is associated with a decrease in glutathione peroxidase activity.

Inconsistently with a number of reported data in animals and adult humans, no associations were found by Blasig et al. [26] between selenium levels and any thyroid hormones. In this cross-sectional analysis of eighty-four children diagnosed with congenital hypothyroidism and a wide range of thyroid hormone concentrations, the serum levels of selenium were measured. The concentrations obtained for Se (mean ± SD) were 69.2 ± 17.4 μg/L, and ranged from 33 to 148 μg/L. The authors considered <70 μg/L to be an indicator of Se deficiency, and <20 μg/L to be an indicator of severe deficiency. Half of the children in the study showed a selenium deficiency. A major limitation of the study was the study design which did not allow testing for causal interactions nor for longitudinal consequences, and the missing hormone values of some of the children. Therefore, the conclusions on the lack of associations between selenium levels and thyroid hormones are not fully convincing. Furthermore, another study by Benamer et al. [25] determining Se concentrations in whole blood revealed no significant differences between patients with hypothyroidism and the healthy controls. However, this study was based on an extremely small sample size (*n* = 8 hypothyroid patients), thus it was difficult to draw reliable conclusions.

Nourbakhsh et al. [37] evaluated serum Se and SePP concentrations as well as glutathione peroxidase (GPx) activity in erythrocytes of adolescents and children with hypothyroidism (22) and healthy controls (30). The results indicated that selenium concentrations in patients with hypothyroidism were comparable to the levels determined in the control group. A study by Verni et al. [44] established multielemental and metalloprotein profiles for two groups using a multivariate principal components model. The study was conducted on 20 patients with hypothyroidism and 20 healthy people. In this study as well, there were no significant differences in selenium concentration between groups (49.3 ± 14.6 vs. 54.1 ± 11.13 μg/L). Liu et al. [52] measured the contents of chemical elements, including selenium, in the erythrocytes of 12 hypothyroid and 20 healthy subjects. For selenium no significant differences were observed between the group with hypothyroidism and healthy subjects (*p* < 0.05). Cayir et al. [27] investigated the association between levels of trace elements, including selenium, and the occurrence of subclinical hypothyroidism in obese patients. In a sample of 109 children (28 overweight, 29 obese, 28 morbidly obese, 24 controls), there was no significant correlation between serum thyroid hormones and levels of trace elements. The levels of FT4 and Se were significantly affected in exogenous obesity patients compared to controls. However, the change in FT4 levels was not correlated with changes in the concentrations of selenium.

Low Se concentration was associated with progressive intensification of autoimmune processes and development of Graves’ disease, autoimmune thyroiditis and thyroid cancer [56]. Adequate selenium levels are important in initiating a normal immune response or regulating its intensity and in reducing chronic inflammation.

A high selenium level significantly increases the proliferation capacity of CD4+ T cells into T regulatory cells. A reduction in or disturbance of Treg activity may lead to the development of an autoimmune disease. Selenium increases the activity of Treg cells while suppressing the release of cytokines, and protects the thyroid follicular cells against apoptosis [11].

The SERENA study (2019) demonstrated that selenium supplementation at 83 mcg/day during pregnancy and after delivery does not pose any risk but has a beneficial effect on auto-antibody titer and on postpartum thyroiditis recurrence [59].

Erdal et al. [28] conducted a study on a group of 43 autoimmune thyroiditis patients with subclinical hypothyroidism (SCH) before and after thyroid replacement therapy and 49 healthy controls living in an iodine-rich area in Turkey. The serum concentration of Se was evaluated. The effects of thyroid hormone replacement therapy on other laboratory and clinical parameters (BMI, FT3, FT4, TSH, total cholesterol, triglycerides, LDL and HDL cholesterol, CRP, and homocysteine) in SCH patients were also controlled. Concentrations of selenium in patients with SCH before treatment with levothyroxine were 67.7 ± 10.4 mmol/L, whereas concentrations after treatment were 66.2 ± 15.7 mmol/L. In the control group the serum levels of selenium were significantly higher (83.7 ± 17.3 mmol/L). A negative correlation between Se levels and hsCRP was observed. In patients with levels of Se < 80 μg/L (*n* = 31), hsCRP levels were significantly higher than in patients with Se >80 μg/L. At the end of the study, the authors observed no association between thyroid function and concentrations of selenium, and no significant changes in BMI, cholesterol levels, homocysteine, hsCRP, vitamin B_12_, or folate levels. However, Se deficiency, which is prevalent in autoimmune thyroiditis patients with SCH, may increase the risk of cardiovascular disease, due to the antioxidant role of Se.

Federige et al. [29] tested the hypothesis that Se concentration is significantly different in patients with a thyroid disorder compared with controls. Serum Se and selenoprotein P (SePP) values were measured in 73 patients with autoimmune thyroid disorders (54 female and 19 male) and 27 healthy subjects living in Brazil. The patients were divided into two groups—Hashimoto’s thyroiditis (HT) without LT4 treatment (*n* = 14) and HT with LT4 treatment (*n* = 19). Similar median (range) concentrations were recorded: 51.9 (44.6–58.5 μg/L), 54.4 (44–63.4 μg/L), 56.0 (52.4–61.5 μg/L) in HT without LT4 treatment, HT with LT4 treatment and controls, respectively (*p* = 0.48). Serum SePP levels were lower (*p* = 0.002) in HT without LT4 treatment patients compared to the control group: 0.35 (0.2–1.17 μg/mL) vs. 1.00 (0.56–4.21 μg/mL). There was a significant correlation between SePP and TPOAb levels. However, there were no variables related to Se or SePP. A low serum selenoprotein P concentration in HT patients may lead to inflammatory reactions.

A study by Rostami et al. [40] was undertaken to assess the relationships linking serum Se concentration with parameters describing thyroid function and antioxidant defense in HT patients with hypoechogenic thyroid. The study included 49 newly diagnosed patients with HT and 50 healthy controls. The results indicate that HT patients had lower Se concentrations than controls (*p* < 0.001). The frequency of Se deficiency was 58.8% and 34% in Hashimoto’s thyroiditis and controls, respectively. In the HT patients, Se deficiency was associated with increased whole-body iodine store, TVol, TPO-Ab, GPx3 activity, and TSH compared to Se-sufficient patients. The authors suggest that Se deficiency and elevated iodine in HT may lead to enhanced autoimmune reactions and accelerate the deterioration of thyroid function through oxidative stress [40].

Another study by Zagrodzki et al. [45] assessed the associations between parameters expressing Se status, thyroid function, and secretion of sex hormones. The correlations were examined in a group of 30 women (15 with HT and 15 healthy subjects). Levels of THS and anti-TPO were higher in patients with Hashimoto in comparison to healthy subjects (2.7 vs. 1.4 μIU/L and 390 vs. 5.7 U/L, respectively). Activity of GPX3 was lower in patients with Hashimoto’s than in controls (248.7 vs. 268.0 U/L). The study confirmed the association between parameters. The authors suggest that the selenoenzyme glutathione peroxidase (GPX3) and progesterone are the most significant factors influencing the regulation of thyroid-stimulating hormone and free thyroxine. In addition, they observed a correlation between GPX3 and estradiol, which is consistent with results reported by other authors. Patients with Hashimoto’s disease showed lower glutathione peroxidase activity than healthy subjects [45]. Selenium deficiency in pregnant women is associated with an increased risk of preterm labor, preeclampsia, miscarriage, gestational diabetes, and other obstetric complications.

Study by Ambroziak et al. [24] examined the hypothesis that the decline in selenium status during pregnancy may be exacerbated in patients with autoimmune thyroid disease (AITD). Selenium status was determined by two biomarkers: serum selenium and selenoprotein P concentrations. The results taken from 29 pregnant women with AITD were compared to results from the European Prospective Investigation into Cancer and Nutrition (EPIC) study. Serum Se and selenoprotein P concentrations were relatively low in AITD women and healthy controls. In AITD patients, TG-aAb and TPO-aAb levels declined during pregnancy by 60% and 71%, respectively, but it was not associated with Se status. There were no pregnancy complications in AIDT or control groups. Newborn TSH was also measured, and the results showed no significant changes in average TSH levels in children born to mothers with AITD (1.4 ± 1.4 mIU/L) in comparison to children born to mothers from the control group (1.8 ± 1.4 mIU/L). There were no associations between newborn TSH levels and the mother’s serum Se or selenoprotein P concentration [24].

Krassas et al. conducted a study in which plasma selenium status and urine iodine levels were measured in a sample of 160 Hashimoto’s thyroiditis patients and 27 control subjects from four European countries [49]. To assess Se status, two meaningful plasma biomarkers were used, i.e., total p-Se and p-SePP. A linear correlation was found between plasma Se and plasma SePP, which explains the less than optimal Se concentration in thyroid patients. The authors suggest that Se supplementation might have a beneficial effect in autoimmune thyroid patients.

Nourbakhsh et al. evaluated serum Se and SePP concentrations as well as glutathione peroxidase (GPx) activity in the erythrocytes of adolescents and children with HT (35) and healthy controls (30). The results showed no significant differences in serum selenium levels between patients and healthy subjects [37]. SePP levels and GPx activity, which are markers of Se status, were also similar in all individuals. There were no associations between GPx or SePP and TPOAb or TgAb.

Stojsavljević et al. published a paper [47] in which concentrations of a variety of trace elements. including selenium, were examined. The levels of the elements were measured in thyroid tissue, blood and urine in HT patients. Pb was negatively correlated with Se, which may be explained by the antagonistic effect of Pb on extrusion of Se from tissue. Se levels in the blood were reduced, while Se content in urine was increased, which additionally explains the lack of Se in HT.

A study by Przybylik-Mazurek et al. investigated the associations between concentrations of Se and thyroid function, parameters of redox balance, and clinical data such as levothyroxine dosage, illness and therapy duration [38]. The participants were a group of 17 females with Hashimoto’s disease, of which eight had been treated previously, and nine were newly diagnosed. The results taken from patients were similar to the control group. There were no differences in concentrations of trace elements or any other antioxidant status parameter between subjects depending on thyroid hormone status (euthyroid, hypothyroidism, or subclinical hypothyroidism). Similarly, treatment with LT4 had no influence on these parameters. 

### 3.2. Zinc

Zinc (Zn) plays a key role in several physiological and biochemical processes in living organisms. It is the essential trace element for energy metabolism. Zn functions as a cofactor for over 300 metalloproteins, such as alcohol dehydrogenase, carbonic anhydrase and alkaline phosphatase, and thus is involved in lipid, protein and carbohydrate metabolism. This mineral is needed for the functioning of the endocrine (particularly thyroid hormone metabolism) and immune systems, and is also involved in DNA transcription as an integral component of zinc finger proteins [60]. In the endocrine system, zinc is required for the receptor activity of thyroid hormones (especially T3) and for conversion of thyroxine to triiodothyronine, and it affects levels of T4 by increasing the production of thyroxin-binding protein [16]. The Recommended Dietary Allowance (RDA) for adults is 8 mg/day for women and 11 mg/day for men [61]. There is no research showing a connection between zinc deficiency and hypothyroidism in humans. In experimental hypothyroidism in a murine model, decreasing concentrations of circulating TH were associated with a significant Zn loss. This process was reversible by Zn replacement, which was associated with restoration of T lymphocyte function [62]. This is the only documented association between zinc concentration and hypothyroidism development. The subject requires more research.

On the other hand a cross-sectional study of 323 patients [34] assessed the status of trace elements, including Zn, in thyroid patients. The subjects were categorized into two groups depending on the type of diagnosis: hypothyroidism (*n* = 61) and autoimmune thyroiditis (*n* = 107). There were significant differences in serum concentrations of Zn between patients and controls. Serum Zn concentrations were relatively low as compared to the group of healthy subjects, but the majority of subjects were within the reference range of 12–16 μM Zn (equal to 0.785–1.046 mg/L). There was no relationship between the type of thyroid disease and a pronounced zinc deficiency [34]. 

The aim of a study by Maouche et al. [50] was to investigate the associations between zinc and oxidative stress markers (total antioxidant status (TAS), glutathione peroxidase (GPx)*,* and superoxide dismutase (SOD)) as well as indices of thyroid hormone disorders (TSH, FT4, FT3, Anti-TPO-Ab) and insulin resistance syndrome (IRS) in the participants classified into two untreated groups: subclinical hypothyroidism (SCH) and overt hypothyroidism (OH). There were no correlations linking Zn with plasma SOD activity. The authors observed that the pathophysiology of dysthyroidism is linked to thyrotropic axis disturbance and also to antioxidant disturbances. Antioxidant trace elements are involved in modulation of oxidative stress in the thyroid gland.

Khatun et al. [33] determined the serum zinc concentrations in patients with hypothyroidism and assessed its impact on the progression of the disease. The levels of the elements were measured in 80 hypothyroid patients and 80 controls. The authors observed that Zn levels were significantly lower in hypothyroid subjects than in controls. Zn was positively correlated with FT3 and negatively correlated with TSH.

Liu et al. [52] measured the contents of chemical elements, including Zn, in the erythrocytes of 12 hypothyroid and 20 healthy subjects. Zinc levels were lower in the group of patients with hypothyroidism than in the control group. In the hypothyroid group, there were relatively strong correlations between Zn and the T3/T4 ratio and between Zn and TSH. Razaei et al. [39] found that hypothyroidism was associated with low concentrations of Zn. Nisa et al. [36] determined the concentration of zinc in subjects with hypothyroidism and assessed the correlation between this metal and thyroid hormones. There was no significant difference between the level of Zn in patients with hypothyroidism and the group of healthy subjects (12.19 ± 3.33 vs. 12.99 ±2.7). The authors observed a strong negative correlation between Zn levels and thyroid hormone levels. Cayir et al. [27] investigated the association between levels of Zn and the occurrence of subclinical hypothyroidism in obese patients. In a sample of 109 children (28 overweight, 29 obese, 28 morbidly obese, 24 controls), there was no significant correlation between serum thyroid hormones and levels of Zn, however, the levels of Zn were significantly affected in exogenous obesity patients compared to controls. The change in FT4 levels was not correlated with changes in the concentrations of Zn. Hanif et al. [31] published a paper in which the correlations between selected trace elements and thyroid hormones were studied. The study was conducted on 68 patients with hypothyroidism and 68 healthy subjects. The majority of the subjects were women, 79% in the group with hypothyroidism and 73% in the control group. In addition to sex and health status, the analysis took into account the type of diet (vegetarian and non-vegetarian) and tobacco use. The mean levels of Zn in the controls were higher than in the hyperthyroid patients but lower than in the hypothyroid patients. There were some differences in the concentrations of most analyzed trace metals depending on sex, cigarette smoking and eating habits. The mean serum concentrations of metals differed between healthy donors and patients with hypothyroidism, indicating that the distribution of trace metals was significantly influenced by the health of the subjects. Stojsavljević et al. [43] published a paper in which the concentrations of trace elements (including Zn) were examined. Serum levels of elements were measured in 93 patients (23 with hypothyroidism and 70 healthy subjects). The concentrations of Zn were higher in the group of respondents suffering from hypothyroidism.

Erdal et al. [28] conducted a study on a group of 43 autoimmune thyroiditis patients with subclinical hypothyroidism (SCH) before and after thyroid replacement therapy and 49 healthy controls. Concentrations of Zn in patients with SCH before treatment with levothyroxine were 109.3 ± 34.3 mmol/L, and concentrations after treatment were 103.6 ± 35.3 mmol/L. In the control group the serum levels of Zn were Zn—101.5 ± 10.7 mmol/L. Significant differences were observed in the concentrations of Zn between the patients and the control group, but the authors observed no association between thyroid function and concentrations of Zn, and no significant changes in BMI, cholesterol levels, homocysteine, hsCRP, vitamin B_12_, or folate levels. Stojsavljević et al. published a paper [47] in which concentrations of a number of trace elements, including Zn, were examined in thyroid tissue, blood and urine in HT patients. In all analyzed thyroid tissue samples the level of Zn was the highest, and the content of other elements was as follows: copper (Cu) > selenium (Se), manganese (Mn) > lead (Pb) > cadmium (Cd) > arsenic (As) > uranium (U). Differences were observed in the elemental profile between HT and healthy subjects. A study by Przybylik-Mazurek et al. [38] investigated the associations between concentrations of Zn and thyroid function, parameters of redox balance, and clinical data such as levothyroxine dosage, illness and therapy duration. The participants were a group of 17 females with Hashimoto’s disease, of which eight had been treated previously, and nine were newly diagnosed. The results taken from patients were similar to the control group. There were no differences in concentrations of Zn or any other antioxidant status parameter between subjects depending on thyroid hormone status (euthyroid, hypothyroidism, or subclinical hypothyroidism). Similarly, treatment with LT4 had no influence on these parameters. 

### 3.3. Copper

Copper, one of the most abundant minerals in the human body, plays an important role in thyroid metabolism, especially in hormone production and absorption. Copper regulates the production of T4 and modulates absorption of T4 in the blood cells by controlling the body’s calcium levels.

In a cross-sectional analysis of eighty-four children diagnosed with congenital hypothyroidism and a wide range of thyroid hormone concentrations, Blasig et al. [26] measured the serum levels of copper. The Cu concentrations obtained (mean ± SD) were 1384.2 ± 388.8 μg/L, and ranged from 748 to 2588 μg/L, which the authors considered to be a normal range consistent with the latest literature. There was a strong positive correlation between Cu levels and thyroid hormones. The authors suggest that the positive correlation of thyroid hormones with copper concentration requires attention. Children with severe hypothyroidism as a result of the disease may develop Cu deficiency as an additional factor disrupting normal development, possibly resulting in growth retardation, growth failure, or other health hazards. The study showed a close interaction of the thyroid axis with copper metabolism in children with primary hypothyroidism. According to the authors, determination of copper levels can be used as an additional, independent biomarker for assessing the effectiveness of thyroid hormone replacement therapy in congenital hypothyroidism.

Another cross-sectional study of 323 patients assessed the status of selected trace elements, including copper, in thyroid patients [34]. The subjects were categorized into two groups depending on the type of diagnosis: hypothyroidism (*n* = 61) and autoimmune thyroiditis (*n* = 107). Serum levels of copper were similar between the groups. 

The aim of a study by Maouche et al. [50] was to investigate the associations between trace elements, including copper, and oxidative stress markers (total antioxidant status (TAS), GPx, and SOD) as well as indices of thyroid hormone disorders (TSH, FT4, FT3, Anti-TPO-Ab). The participants were classified into two untreated groups: subclinical hypothyroidism (SCH) and overt hypothyroidism (OH). Levels of Cu were not altered in the hypothyroid groups compared to controls. There were no correlations linking Cu or Zn with plasma SOD activity. The authors observed that the pathophysiology of dysthyroidism is linked to thyrotropic axis disturbance and also to antioxidant disturbances. Antioxidant trace elements are involved in modulation of oxidative stress in the thyroid gland.

Khatun et al. [33] determined the serum copper concentrations in patients with hypothyroidism and assessed its impact on the progression of the disease. The levels of the element were measured in 80 hypothyroid patients and 80 controls. The Cu concentrations (151.1 μg/dL) were higher than among controls (140.5 μg/dL), but the difference was not statistically significant. It is commonly believed that reduced zinc levels lead to an increase in copper absorption from the gut. There was a positive correlation between Cu and FT3, and FT4, as well as a negative correlation between Cu and TSH. There was also a positive correlation between the FT3/FT4 ratio and the Cu/Zn ratio in the group of patients. The results suggest that the FT3/FT4 ratio is a more useful biomarker to differentiate the causes of chronic thyroid diseases than these parameters individually. According to the authors, the Cu/Zn ratio is a marker of inflammation and is also much more specific in the diagnosis of chronic thyroid diseases than the levels of these elements individually. 

Liu et al. [52] measured the contents of selected chemical elements, including copper, in the erythrocytes of 12 hypothyroid and 20 healthy subjects. No significant differences were observed between the group with hypothyroidism and healthy subjects (*p* < 0.05) for copper.

Razaei et al. [39] found that hypothyroidism was associated with low concentrations of Cu. Cayir et al. [27] investigated the association between levels of a variety of trace elements, and the occurrence of subclinical hypothyroidism in obese patients. In a sample of 109 children (28 overweight, 29 obese, 28 morbidly obese, 24 controls), there was no significant correlation between serum thyroid hormones and levels of copper. The levels of Cu were significantly affected in exogenous obesity patients compared to controls. However, the change in FT4 levels was not correlated with changes in the concentrations of trace elements. 

Hanif et al. [31] published a paper in which the correlations between selected trace elements and thyroid hormones were studied. The study was conducted on 68 patients with hypothyroidism and 68 healthy subjects. The study showed significantly increased levels (mean ± SD) of Cu (2397 ± 0.148 μg/g vs. 1718 ± 0.123 μg/g) in the hypothyroidism group compared to the control group. There were no significant correlations between metals and the hormones. There were some differences in the concentrations of most analyzed trace metals depending on sex, cigarette smoking and eating habits. The mean serum concentrations of metals differed between healthy donors and patients with hypothyroidism, indicating that the distribution of trace metals was significantly influenced by the health of the subjects.

Stojsavljević et al. [43] published a paper in which the concentrations of trace elements, including copper, were examined. Serum levels of elements were measured in 93 patients (23 with hypothyroidism and 70 healthy subjects). The concentrations of copper were higher (*p* < 0.0001) in the group of respondents suffering from hypothyroidism. The results indicated clear differences in element profiles between hypothyroidism and healthy subjects, indicating that this could be used as a unique profile of hypothyroidism.

Erdal et al. [28] conducted a study on a group of 43 autoimmune thyroiditis patients with subclinical hypothyroidism (SCH) before and after thyroid replacement therapy and 49 healthy controls. Concentrations of trace elements in patients with SCH before treatment with levothyroxine were 108.1 ± 53.08 (mmol/L), and concentrations after treatment were 118.6 ± 46.5 (mmol/L). In the control group the serum levels of Cu were 106.9 ± 14.9 (mmol/L). Significant differences were observed in the concentrations of trace elements between the patients and the control group; however, the authors reported no association between thyroid function and concentrations of trace elements, and no significant changes in BMI, cholesterol levels, homocysteine, hsCRP, vitamin B_12_, or folate levels.

A study by Przybylik-Mazurek et al. [38] investigated the associations between concentrations of Cu and thyroid function, parameters of redox balance, and clinical data such as levothyroxine dosage, illness and therapy duration in 17 females with Hashimoto’s disease, of which eight had been treated previously, and nine were newly diagnosed. There were no differences in concentrations of trace elements or any other antioxidant status parameter between subjects depending on thyroid hormone status (euthyroid, hypothyroidism, or subclinical hypothyroidism). Similarly, treatment with LT4 had no influence on these parameters. 

### 3.4. Iron

Iron homeostasis is closely linked to the thyroid gland function. Active T3 has been found to modulate hepatic ferritin expression. Hypothyroidism can reduce hydrochloric acid production and impair iron absorption. Thyroid hormones stimulate erythropoiesis- the production of red blood cells. Reduced circulating thyroid hormones T4 and T3 results in reduced red blood cell levels contributing to anaemia. The thyroid peroxidase enzyme (TPO) is a heme-containing enzyme and therefore its activity is dependent on iron. In iron deficiency, there is reduced TPO activity and therefore reduced thyroid hormone production.

Khatiwada et al. [32] conducted a cross-sectional study to examine the association between iron status and thyroid function in school children in Nepal. Urinary iodine concentration, FT3, FT4, TSH, hemoglobin, serum Fe and total iron binding capacity, and percentage transferrin saturation were analyzed. The study group consisted of 183 euthyroid, 3 overt hypothyroid, and 37 subclinical hypothyroid children. Among the children, 80 were anemic, 99 were iron-deficient, and 45 had urinary iodine excretion <100 μg/L. The results indicate that hypothyroidism (subclinical and overt) was common in iron-deficient and anemic children. The relative risk of hypothyroidism was higher in both iron-deficient children and anemic children, as compared to iron-sufficient and non-anemic children. There was a significant negative correlation between TSH and hemoglobin, and transferrin saturation. The study showed a high prevalence of thyroid dysfunction, anemia and iron deficiency among Nepalese children, and a significant relationship between iron levels and thyroid function. Thus, anemia and iron deficiency appear to be related to thyroid dysfunction, especially hypothyroidism.

Liu et al. [52] measured the contents of selected chemical elements, including Fe, in the erythrocytes of 12 hypothyroid and 20 healthy subjects. No significant differences were observed between the group with hypothyroidism and healthy subjects for Fe.

Hanif et al. [31] published a paper in which the correlations between Fe and thyroid hormones were studied. The study was conducted on 68 patients with hypothyroidism and 68 healthy subjects. The study showed significantly increased levels (mean ± SD) of Fe (39.80 ± 3069 μg/g vs. 14.32 ± 1970 μg/g) in the hypothyroidism group compared to the control group. There were no significant correlations between metals and the hormones.

Erdal et al. [28] conducted a study on a group of 43 autoimmune thyroiditis patients with subclinical hypothyroidism (SCH) before and after thyroid replacement therapy and 49 healthy controls. Concentrations of Fe in patients with SCH before treatment with levothyroxine were as 55.7 ± 38 mmol/L, whereas concentrations after treatment 73.2 ± 54.7 mmol/L. In the control group the serum levels of Fe were Fe—75.7 ± 24 mmol/L. Significant differences were observed in the concentrations of Fe between the patients and the control group. Basal levels of Fe were significantly lower in patients than in controls. At the end of the study, the authors observed no association between thyroid function and concentrations of Fe, and no significant changes in BMI, cholesterol levels, homocysteine, hsCRP, vitamin B_12_, or folate levels.

### 3.5. Manganese

Manganese (Mn) is an essential element involved in a number of physiological processes in mammals. It plays a role in endocrine function, immune function, hematopoiesis, and oxidative stress regulation [63]. Mn is a key component of some enzymes, such as manganese superoxide dismutase (Mn-SOD), oxidoreductases, transferases, lyases, hydrolases, isomerases, glutamine synthetase, and ligases. Manganese may have an impact on thyroid hormone homeostasis and neurodevelopmental processes as a result of both direct dysregulation at the level of the thyroid gland and thyroid hormones, or indirectly via modification of dopaminergic control of the thyroid gland and thyroid hormones [18]. There are signs of elevated levels of Mn in a subgroup of patients with autoimmune hypothyroidism, which might indicate altered Mn status in hypothyroidism. Mn does not directly modulate TSH secretion via the dopaminergic pathway. High levels of Mn are known to reduce thyroid hormones and elevate TSH concentration. However, the results should be interpreted with caution [64]. A deficiency of manganese can be involved in some disease susceptibilities, such as poor bone formation and skeletal defects, altered carbohydrate and lipid metabolism, and abnormal glucose tolerance. However, exposure to high concentrations of manganese is potentially toxic and can result in a neuropathy known as manganism [18]. 

Cayir et al. [27] investigated the association between levels of selected trace elements, including Mn, and the occurrence of subclinical hypothyroidism in obese patients. In a sample of 109 children (28 overweight, 29 obese, 28 morbidly obese, 24 controls), there was no significant correlation between serum thyroid hormones and levels of Mn. The levels of FT4, and Mn, were significantly affected in exogenous obesity patients compared to controls. However, the change in FT4 levels was not correlated with changes in the concentrations of Mn. 

In a study by Hanif et al. [31], no significantly elevated manganese levels were observed in this study. A cluster analysis revealed clusters of Fe-Cr, Cd-Pb, Cu-Co-Mn, Zn-TSH and Ni-T4. Chromium shared a cluster with a negligible metal, Fe, indicating an imbalance of these metals in the serum of hypothyroid patients.

Memon et al. [35] conducted a study to compare the serum concentration of Mn with thyroid functions (TSH, FT3, FT4) of hypothyroid patients and controls. The data indicate that the Mn concentration in the serum samples of women with hypothyroidism was significantly lower than in the control group. In patients with hyperthyroidism, there was a negative correlation between serum Mn levels and TSH, while FT3 and FT4 were positively correlated with Mn. In hypothyroid patients, the reverse pattern for correlations of Mn and thyroid hormonal function was observed.

Maouche et al. [50] also measured manganese levels (60 subjects with hypothyroidism (OH), 50 with subclinical hypothyroidism (SCH)). Levels of Mn were increased in the OH and SCH groups compared to controls. Abnormal levels of manganese could be used as an indicator of thyroid oxidative damage in patients predisposed to dysthyroidism. There was a correlation between thyroid hormone disorders (especially the TSH variant) and the status of manganese. The results indicate that a lack of manganese bioavailability leads to a decrease in mitochondrial superoxide dismutase activity.

Stojsavljević et al. [43] examined the levels of Mn. A study conducted on a group of 23 patients with hypothyroidism and 70 healthy subjects showed elevated levels of Mn in patients with hypothyroidism.

Stojsavljević et al. published also a paper [47] in which concentrations of selected trace elements, including Mn were examined. Differences were observed in Mn profile between HT and healthy subjects.

### 3.6. Chromium

Chromium is an essential element that influences carbohydrate, lipid and protein metabolism. Cr(III) increases insulin sensitivity by activating insulin receptor kinase. Since its deficiency can cause an imbalance of the blood sugar levels the adrenal glands may be affected. Consequently, this can lead to thyroid disorders including hypothyroidism. Furthermore, problems with the adrenal glands may in turn affect the immune system, which can potentially trigger an autoimmune condition such as Hashimoto’s Thyroiditis. Therefore direct or indirect influence of Cr (III) on thyroid condition need to be elaborated thoroughly. Trivalent chromium and hexavalent chromium species show different chemical characteristics and biological effects.

Although chromium has been reported to be an essential nutrient, exposure to high levels of Cr(III) via inhalation, ingestion, or dermal contact may cause some adverse health effects [65]. The impact of Cr(III) excess on thyroid gland is relatively unexplored. A similar lack of research applies to a link between highly toxic hexavalent form of chromium and thyroid structure and function. In part, this may be due to the properties of Cr(VI), which undergoes reduction to Cr(III) after penetration of biological membranes and in the gastric environment [66]. However, the reduced form of chromium is much less able to cross cell membranes than oxidized form of the element. Cr (III) binds directly to transferrin, an iron-transporting protein in the plasma. The reduction process may undergo inside of cells, or outside of cells [66]. Recent studies provide the suggestions that mainly vitamins E and C and Se demonstrate protective effect in occupational groups that are particularly exposed to Cr(VI). However, more research in this area is required [67].

Studies on the immunotoxic effects of Cr(VI) in humans are very rare [68], and a clear relationship between chromium (VI) exposure and the occurrence of hypothyroidism over the last twenty years has not been established. There is still a need to clarify the mechanism of an endocrinological activity of chromium especially in conjunction with the immune system. Animal studies conducted by Mahmood et al. [69] suggest that thyroid is sensitive to various chemical forms of chromium and that exposure to chromium (VI) can result inter alia in oxidative changes in proteins [70].

So far, most of the published research into endocrine effects of chromium regards the reduced form of this element. In a study by Hanif et al. [31] significantly increased levels of chromium were observed in patients with hypothyroidism compared to the control group (Cr 12.31 ± 1.229 μg/g vs. 8.422 ± 0.886 μg/g). Rezaei et al. [39] found that elevated levels of Cr were linked to an increased risk of hypothyroidism.

A study conducted by Stojsavljević et al. [43] on a group of 23 patients with hypothyroidism and 70 healthy subjects showed elevated levels of Cr these in patients with hypothyroidism. However, Verni et al. [44] observed that Cr concentration in the control group was from 2 to 3 orders of magnitude higher than in patients with hypothyroidism, which is not consistent with the current state of knowledge.

Stojsavljević et al. [47] observed the differences in the elemental profile between HT and healthy subjects. According to the research into the influence of Cr on thyroid gland function serum Cr concentrations have been noted to correlate well with thyroid gland activity and high serum chromium levels have been reported in hyperthyroidism.

Findings of Hasan et al. studies [71] demonstrate that serum thyroid peroxidase activity and chromium levels were significantly higher in hyperthyroidism patients (152.0 ± 4.6 IU/L) (2.159 ± 0.15 ppb) than normal (51.0 ± 1.8 IU/L) (0.378 ± 0.024 ppb) respectively and lower than normal in hypothyroidism patients (35.0 ± 0.31 IU/L) (0.099 ± 0.011 ppb) respectively.

### 3.7. Fluoride

Malin et al. [51] conducted the first population study examining the effects of chronic exposure to low doses of fluoride on thyroid function. He interpreted the levels of fluoride in relation to iodine levels. The study was intended to determine whether urinary iodine status modifies the effect of fluoride exposure on thyroid stimulating hormone (TSH) levels. The study population consisted of 6,914,124 adults in Canada aged 18–79 who were not taking any thyroid-related medication (1,346,329 adults with moderate/severe iodine deficiencies and 5,567,795 adults without moderate/severe iodine deficiencies). Urine fluoride concentrations were analyzed in spot samples using an ion selective electrode. In order to take into account differences in urine dilution, urinary fluoride concentrations were adjusted for specific gravity (UF_SG_; mg/L). Thyroid function was assessed on the basis of TSH levels. Approximately 17.8% of the participants had moderate to severe iodine deficiency. The median concentration of UF_SG_ was 0.74 mg/L. In iodine-deficient adults, an increase in UF_SG_ of 1 mg/L was associated with an increase in TSH of 0.35 mIU/L (95% CI: 0.06, 0.64; *p* = 0.01, one-tailed). The study suggests that adults with moderate to severe iodine deficiency and higher levels of urinary fluoride may be at increased risk of developing hypothyroidism.

Previously, Peckhman et al. [72]], in the data from a cross-sectional study on fluoride levels in drinking water and the number of diagnosed cases of hypothyroidism among the population of England, found that assessment of fluoride levels in drinking water can be used to predict the occurrence of hypothyroidism in a given area. It was observed that in the fully fluorinated area, hypothyroidism was reported almost twice as often as in the non-fluorinated area.

Singh et al. [42] conducted a study on a group of 60 children aged 8–15 years living in the endemic fluoride area of the Udaipur district. Children were divided into two groups: 30 with dental fluorosis and 30 without dental fluorosis. The control group consisted of 10 children from the non-endemic area. The aim of the study was to determine the levels of fluoride in urine and serum and to determine relationships between the concentrations and levels of FT4, FT3 and TSH. The study showed statistically significant relationships between fluoride in drinking water and both urine fluoride and serum fluoride. Significantly altered levels of FT3, FT4 and TSH hormones, as well as increased levels of fluoride in the serum and urine were observed in children from the study group. A significant relationship was also shown between serum fluoride concentration and the concentrations of thyroid hormone (FT3/FT4) and TSH. According to the authors, the results undermine the validity of fluoridation of drinking water and food products. A similar study conducted by Xiang et al. [73] came to the same conclusions. However, in a study by Hosur et al. [74], no significant changes in the levels of FT3, FT4 and TSH thyroid hormones were observed in people with dental fluorosis from endemic fluorosis populations compared to controls (10 subjects without dental fluorosis).

Kharedpisheh et al. [19] conducted a study of the effect of fluoride in drinking water on T3, T4 and TSH levels of people living in Yazd Greater Area in Iran. This area is characterized by the presence of drinking water wells with varied levels of fluoride. The spline GIS model was used to determine the level of fluoride in drinking water samples. None of the samples contained fluoride with a concentration higher than the upper limit of the global standard (0.5–1.5 mg/L). The study included 198 people with hypothyroidism and 213 healthy people. The average levels of TSH and T3 hormones was statistically significantly associated with fluoride levels, which were analyzed in concentrations of 0–0.29 mg/L and 0.3–0.5 mg/L (*p* = 0.001 for controls and *p* = 0.001 for cases). Among those with untreated hypothyroidism, as well as in the control group, TSH values were higher at higher fluoride concentrations in drinking water, even if fluoride concentrations were low. The study demonstrated that fluoride affects TSH and T3, even at a standard concentration below 0.5 mg/L. The authors concluded that people with hypothyroidism should use home drinking water treatment devices to reduce the concentration of fluoride.

On the other hand, a study conducted by Shaik et al. [41] did not confirm that fluoride from drinking water affects the concentration and functions of thyroid hormones. In a group of 293 children aged between 9 and 13 years with normal nutritional status and optimal iodine intake, long-term consumption of fluoridated drinking water (0.02–1.4 ppm) had no effect on thyroid function.

### 3.8. Lead (Pb)

Lead is a toxic heavy metal possessing high potential to disturb endocrine system. There are some inconsistent results suggesting that lead might have different etiological roles in the thyroid diseases [75]; on the other hand, investigations of the role of Pb in oxidative—antioxidant imbalance resulting in increased lipid peroxidation and altered antioxidant enzymes are rather consistent [76].

Egyptian studies revealed that workers exposed to Pb dust proved to be at risk for hyperthyroidism (showed significantly decreased TSH mean levels, and a significant increase in FT3 and FT4 mean levels compared to the control group). However, the authors highlighted an inconsistency of results between the associations of Pb with thyroid hormones [77]. The results of meta-analysis conducted by Krieg [78] did not provide evidence for an effect of occupational lead exposure on thyroid function in men. On the other hand, a study suggested that lead exposure can induce functional impairment of the pituitary-thyroid axis and provoke the alteration of TSH levels [79].

Hanif et al. [31] published a study that measured lead levels in 68 people with hypothyroidism and 68 healthy people. Levels of lead in the serum of hypothyroid people were significantly increased (*p* < 0.05) compared to the control group. The blood serum Pb concentrations were 12.84 ± 1.287 μg/g in the hypothyroidism group and 5.365 ± 0.761 μg/g in the control group.

A similar conclusion was also reached by Rezaei et al. [39], who in a group of 110 participants (*n* = 33 with hypothyroidism and *n* = 33 healthy) observed elevated levels of lead and cadmium among patients with hypothyroidism. All patients were newly diagnosed, and at the time of diagnosis were under no specific treatment. The authors noted that elevated levels of cadmium and lead are linked to hypothyroidism development.

In another study, Osterode et al. [46] tested whether patients with hypothyroidism and increased bone metabolism as a result of levothyroxine medication show an increase in Pb excretion. Lead concentrations were measured in whole blood (PbB) and urine (PbU). None of the patients had been occupationally exposed to Pb. The results show that the PbB concentration of the untreated hypothyroid patients did not differ from the controls, but it was slightly lower after levothyroxine treatment (*p* = 0.053). PbU concentrations in the untreated and treated subjects did not differ from the controls, but after levothyroxine medication Pb excretion was increased. There were highly significant correlations between PbU and FT3, and T4. There were no correlations between PbB and thyroid hormones (FT4 and FT3). The data indicate an increase in Pb excretion after levothyroxine therapy.

Stojsavljević et al. published a paper [47] in which concentrations of selected trace elements, including Pb, were examined. One of the most important findings in the study were elevated Pb concentrations in the thyroid tissue and blood samples of Hashimoto’s thyroiditis patients. Pb was negatively correlated with Se, which may be explained by the antagonistic effect of Pb on extrusion of Se from tissue. Se levels in the blood were reduced, while Se content in urine was increased, which additionally explains the lack of Se in HT.

### 3.9. Cadmium

Cadmium has a strong toxic effect on the thyroid gland [80]. However, the precise mechanisms underlying the effects of Cd as an endocrine disruptor remain to be elucidated [81]. It has been shown that the thyroid gland of people exposed to cadmium accumulates 3 times more cadmium compared to that of unexposed people [82]. The clinical significance from Cd-exposure implies enhanced oxidative stress and mitochondrial dysfunction [83]. The evidence for Cd-thyrotoxicity which can lead to hyperfunction or hypofunction of the thyroid gland has been reported [84].

Hanif et al. [31] published a study that assessed lead levels in 68 people with hypothyroidism and 68 healthy people. Patients showed elevated levels of cadmium: hypothyroid 1.182 ± 0.114 μg/g, controls 0.423 ± 0.035 μg/g. Stojsavljević et al. [43] also observed elevated levels of cadmium among patients with hypothyroidism.

A similar conclusion was also reached by Rezaei et al. [39], who in a group of 110 participants (*n* = 33 with hypothyroidism and *n* = 33 healthy) observed elevated levels of lead and cadmium among patients with hypothyroidism. All patients were newly diagnosed, and at the time of diagnosis were under no specific treatment. The authors noted that elevated levels of cadmium and lead are linked to hypothyroidism development.

Table 2 lists the examples of determinations of selected elements in the research cited above.

### 3.10. Summary of the Findings

Many elements are crucial for the synthesis of thyroid hormones that maintain the proper rate of metabolism; therefore, research on the possible impact of the imbalance of these elements on hypothyroidism is of a high importance. The relationships between the trace elements and the thyroid gland function are mutual. The elements are well known to be important activators or inhibitors of enzymatic reactions in the thyroid gland. On the other hand, the thyroid gland influences metabolism, transport and excretion of the elements. It is not fully understood how many elements possess the significant effect on the thyroid gland function. Our literature review revealed many significant discrepancies to the associations between thyroid function tests and selected serum trace elements. For all the above discussed elements, except lead, the results are highly inconsistent (Table 2).

All the cited studies [45,57,61,63] revealed significantly elevated lead levels in patients with hypothyroidism, including autoimmune thyroiditis (Hashimoto’s disease) in comparison to healthy subjects.

Findings on zinc in patients with hypothyroidism, including patients with Hashimoto’s disease, indicate the possible existence of an association between Zn level in the body and the normal functioning of the thyroid hormone system [42,57,60,61,74,85,86]. Nearly all of the studies that reported a correlation between Zn level in the body and the thyreometabolic status showed a significant zinc deficiency in hypothyroidism [42,57,61,74,85,86]. Since free triiodothyronine and thyroxine require zinc to fulfill their biological activity, a deficiency of this trace element negatively affects the metabolic activity of these both hormones. The possible link between Zn deficiency and hypothyroidism may be attributed to the impairment of gastrointestinal absorption of Zn in hypothyroidism patients. 

Interestingly, the results of one research indicated significantly elevated zinc levels in the hypothyroid group [60], whereas other studies did not indicate any significant differences in zinc levels in patients with hypothyroidism compared to healthy subjects. It should also be emphasized that zinc acts as a modulator of the adaptive immune system’s inflammatory response, and in addition it can inhibit Th17 lymphocytes. It is suggested that Zn can probably help to slow the progression of autoimmune thyroiditis.

Similar discrepancies were found in studies concerning cadmium. Some researches [45,57,60], devoted to the analysis of Cd, showed that Cd is significantly elevated in patients with hypothyroidism. However, one study [61] reported significantly reduced levels of this element in the course of Hashimoto’s disease, whereas other studies showed no significance [74].

Additionally, findings have been equivocal in linking selenium to hypothyroidism. Several reports [43,58,61,86] demonstrated significant selenium deficiency hypothyroidism, however, there are studies [42,60] in which hypothyroidism was associated with a significant increase in selenium serum levels. Furthermore, the larger set of studies [39,40,47,54,64,72,74,87,88] revealed no significant differences in the level of this element in patients with hypothyroidism compared to healthy controls. Although selenium and iodine are among the most intensively studied elements in the wide context of a number of thyroid diseases, some aspects of action of these elements in the thyroid gland still need to be elucidated. Alterations in the pool of some stored elements, such as iodine, selenium, and zinc, in the thyroid gland may affect the function of this organ depending on the secretion of TSH by the pituitary gland, responsible for the regulation of T3 and T4 hormones.

For manganese, chromium, copper, iron and fluorine, the findings are equally inconsistent as the above mentioned examples. More research is required to determine whether the level of these trace elements can affect the level of thyroid hormones. Furthermore, it remains still unknown how the interactions between some of these elements can regulate the thyroid gland function. Interestingly, since the ratio of selenium and copper may affect the synthesis of thyroid hormones, the compelling issue arises as to whether an increased selenium along with a decreased copper levels may induce a reduction in the doses of L-thyroxine, used in the therapy of hypothyroidism.

Complexity of the discussed problem and possible sources of discrepancies may arise from the varied characteristics of the population, and from the adopted research methodologies. The prominent differences in some variables such as age, sex, geographical location, time of exposure, and use of alcohol or tobacco by the subjects often make the comparison of different studies on trace element contents difficult. These factors collectively lead to the perceptional distortion of the issue of possible elemental effects on the thyroid status. The ambiguity of the results of the described studies may erroneously lead to an underestimation of the importance of the role that elements play in hypothyroidism. This may also lead to the underestimation of the risks associated with a deficiency or excess of certain elements in hypothyroidism. Elemental imbalance may exacerbate hormonal imbalance in hypothyroidism that leads to deterioration of the patient’s condition. Undoubtedly, without a complete picture of the elemental status in the body, it is difficult to draw conclusions on possible associations between the imbalance of a particular element and hypothyroidism. For example, Na^+^/I^−^ symporter function can be inhibited or stimulated by various chemicals, including toxic metals, which in turn are able to interact with essential elements. Chemicals, when combined, may interact and exert synergistic or antagonistic effects. Moreover, other chemicals (organic and inorganic compounds), that have thyroid-disrupting potential, may influence the elemental balance. It seems justified to conduct extensive multi-elemental and multi-compound studies to eliminate inaccuracies and inconsistencies of the research. In addition, estimates of associated diseases (e.g., infectious diseases or obesity that affect trace element content in the blood as a result of altered biochemical and metabolic processes) are largely missing, so the quality of available studies may not be sufficient to draw valid conclusions. Information on comorbidities is unfortunately omitted in many papers.

Although the results of many studies confirm the significant effects of individual elements on thyroid disorders, our review indicated that there are significant inconsistencies regarding how studies into the effects of chemical elements on hypothyroidism are conducted.

### 3.11. Strengths and Limitations

The main strength of this article is that it provides a summary of studies based on the measurement of concentrations of selected chemical elements in the context of hypothyroidism. Articles examining the concentrations of chemical elements in hypothyroid patients were included. The main limitations of our review are the inclusion of only articles in English and the lack of access to the full text of some papers.

## 4. Conclusions

Globally, a vast number of people suffer from thyroid-related problems, especially hypothyroidism. Over the past two decades, a number of studies have focused on the effects of environmental toxins on the condition of the human endocrine system, including the impact of selected trace elements (selenium, zinc, manganese, chromium, copper, iron, iodine, fluorine and lead) on the thyroid gland. An overview of published data on hypothyroidism and chemical elements yields inconsistent results. Many of them describe an important connection between concentrations of certain elements and clinical/laboratory parameters of thyroid functions. However, the results are not conclusive. One of the causes may be differences in the analytical methods used as well as in planning research. Moreover, the effect of elemental supplementation before disease onset is usually not examined. Even surveys carried out over many years were unable to clearly establish whether a deficiency or excess of certain chemical elements or exposure to heavy metals can be considered an etiopathogenetic factor of hypothyroidism. A thorough understanding of the influence of metabolism of elements on thyroid hormone metabolism may be useful in planning the treatment or prevention of this illness. Our analysis of scientific databases confirms that knowledge of the role of trace elements, including heavy metals, on thyroid homeostasis will contribute to an understanding of the causes of hypothyroidism development.

## Figures and Tables

**Table 1:** Article inclusion and exclusion criteria.

Inclusion Criteria	Exclusion Criteria
Articles written in English	Articles written in a language other than English
Articles containing original data or meta-analyses	Articles that do not contain original data: reviews, mini-reviews, comments, and letters
Peer-reviewed publications	Publications which have not been independently peer-reviewed
Studies on humans	Studies on animals

**Table 2:** Examples of determinations of selected elements in the research cited above. Data are presented as: a. mean (±SD), b. median (±SD).

Element	Thyroid Disorder (Type of Sample)	Outcome in Patients	Outcome Controls	Significantly Increased (↑) or Decreased (↓) Level in Hypothyroidism Compared with Controls	References
**Fe**	Reference values: serum—50–170 μg/dL^M^; 30–160 μg/dL^W^		Arup Laboratories		[20]
	Reference values: serum—50–150 μg/dL^M^; 35–145 μg/dL^W^		Mayo Clinic Laboratories		[21]
	Hypothyroid (serum)	a. 39.80 ± 3.069 μg/g	a. 14.32 ± 1.970 μg/g	↑	[31]
	Autoimmune thyroiditis patients with SH (serum)	a. Pretreatment: 55.7 ± 38 μg/dL Posttreatment: 73.2 ± 54.7 μg/dL	a. 75.7 ± 24 μg/dL	↓	[28]
	Hypothyroid (serum)	b. 2.659 ± 1.145 μg/ml	b. 8.782 ± 6.792 μg/mL	-	[44]
	Hypothyroid (serum)	a. 475.4 ± 236 μg/g	a. 438.4 ± 186 μg/g	-	[39]
	Hypothyroid (erythrocytes)	a. 2466 ± 314 μg/g Dry Weight	a. 2570 ± 456 μg/g Dry Weight	-	[52]
	Hypothyroid (serum)	a. 57.0 ± 26.6 μg/dL	a. 74.0 ± 36.7 μg/dL	↓	[32]
**Zn**	Reference values: **serum**—60.0–120.0 µg/dL; **urine**—15.0–120.0 μg/dL		Arup Laboratories		[20]
	Reference values: **serum**—0.66–1.10 µg/dL		Mayo Clinic Laboratories		[21]
	Hypothyroid (serum)	a. 9.448 ± 2.124 μg/g	a. 7.944 ± 1.787 μg/g	-	[31]
	Autoimmune thyroiditis patients with subclinical hypothyroidism (serum)	a. Pretreatment: 109.3 ± 34.3 μg/dL Posttreatment: 103.6 ± 35.3 μg/dL	a. 101.5 ± 10.7 μg/dL	-	[28]
	Hypothyroid (serum)	b. 696.7 ± 299.9 μg/L	b. 1865.1 ± 1040.89 μg/L	↓	[44]
	Hypothyroid (serum)	a. 11.7 ± 5.3 μg/g	a. 66.7 ± 49.9 μg/g	↓	[39]
	Hashimoto disease (serum)	a. 12.2 ± 1.4 μM/L	a. 12.5 ± 2.8 μM/L	-	[38]
	Hypothyroid (erythrocytes)	a. 42 ± 13 μg/g Dry Weight	a. 34 ± 10 μg/g Dry Weight	-	[52]
	Hypothyroid (serum)	a. 12.19 ± 3.33	a. 12.99 ± 2.70	-	[36]
	Thyroid patients (serum)	a. 1025.0 ± 232.6 μg/L	a. 1068.0 ± 230.0 μg/L	↓	[34]
	Hypothyroid (serum)	a. 1053.56 ± 113.2 μg/L	a. 548.42 ± 59.48 μg/L	↑	[43]
	Hypothyroid (serum)	a. 85.206 ± 36.112 μg/dL	a. 100.825 ± 23.865 μg/dL	↓	[33]
	Hashimoto thyroiditis (thyroid tissue)	a. 6035 ± 3628 ng/g	a. 6098 ± 2480 ng/g	↓	[47]
	Hashimoto thyroiditis(blood)	a. 1707 ± 243 ng/g	a. 4801 ± 1124 ng/g		
	Hashimoto thyroiditis(urine)	a. 94.3 ± 62.3 ng/g	a. 217 ± 177 ng/g		
	Overweight (serum)	a. Pretreatment: 263.2 ± 91 6th month: 353.3 ± 141	a. 342.3 ± 102	↓	[27]
	Obese(serum)	a. Pretreatment: 292.6 ± 140 6th month: 257.5 ± 132			
	Morbidly obese (serum)	a. Pretreatment: 254.6 ± 125 6th month: 251.5 ± 114			
**Cu**	Reference values:**serum**—70.0–140.0 μg/dL^M^; 80.0–155.0 μg/dL^W^; **urine**—0.3–3.2 μg/dL		Arup Laboratories		[20]
	Reference values: **serum**—0.75–1.45 µg/mL; **urine**—(>18 years) 9–71 mcg/24 h		Mayo Clinic Laboratories		[21]
	Hypothyroid (serum)	a. 2.397 ± 0.148 μg/g	a. 1.718 ± 0.123 μg/g	↑	[31]
	Autoimmune thyroiditis patients with SH (serum)	a. Pretreatment: 108.1 ± 53.08 mmol/ Posttreatment: 118.6 ± 46.5 mmol/L	a. 106.9 ± 14.9 mmol/L	-	[28]
	Hypothyroid (serum)	b. 919.6 ± 365.4 μg/L	b. 454.5 ± 871.67 μg/L	↑	[44]
	Hypothyroid (serum)	a. 29.0 ± 10.4 μg/g	a. 39.2 ± 14.5 μg/g	↓	[39]
	Hashimoto disease (serum)	a. 17.5 ± 2.3 μM/L	a. 17.3 ±3.0 μM/L	-	[38]
	Thyroid patients (serum)	a. 1365.0 ± 372.9 μg/L	a. 1394.0 ± 398.3 μg/L	-	[34]
	Hypothyroid (serum)	a. 151.113 ± 51.015 μg/dL	a. 140.500 ± 21.223 μg/dL	-	[33]
	Hypothyroid (serum)	a. 987.15 ± 246.03 μg/dL	a. 718.26 ± 146.60 μg/dL	↑	[43]
	Hypothyroid (erythrocytes)	a. 9.6 ± 3.8 μg/g Dry Weight	a. 9.6 ± 2.1 μg/g Dry Weight	-	[52]
	Hashimoto thyroiditis (thyroid tissue)	a. 515 ± 398 ng/g	a. 335 ± 153 ng/g	↓	[47]
	Hashimoto thyroiditis(blood)	a. 545 ± 131 ng/g	a. 965 ± 258 ng/g		
	Hashimoto thyroiditis(urine)	a. 132 ± 96.0 ng/g	a. 17.8 ± 3.40 ng/g		
	Congenital hypothyroidism (serum)	a. 1384.2 ± 388.8μg/L	-		[26]
	Overweight (serum)	a. Pretreatment: 229.7 ± 193.7 6th month: 145.3 ± 30.7	a. 172.7 ± 22.9	↑	[27]
	Obese (serum)	a. Pretreatment: 195.2 ± 35.6 6th month: 132.5 ± 37.2			
	Morbidly obese (serum)	a. Pretreatment: 187 ± 50.9 6th month: 147.5 ± 38.7			
**Mn**	Reference values: **serum**—0.0–0.2 μg/L; **blood**—4.2–16.5 μg/L		Arup Laboratories		[20]
	Reference values: **serum**—<2.4 ng/mL; **blood**—4.7–18.3 ng/ml		Mayo Clinic Laboratories		[21]
	Hypothyroid (serum)	a. 0.987 ± 0.103 μg/g	a. 0.899 ± 0.101 μg/g	-	[31]
	Hypothyroid (serum)	b. 50.3 ± 116.5 μg/L	b. 109.0 ± 87.43 μg/L	↓	[44]
	Hypothyroidism (serum)	a. 8.98 ± 3.9 μg/L	a. 0.92 ± 0.25 μg/L	↑	[43]
	Hashimoto thyroiditis (thyroid tissue)	a. 209 ± 125 ng/g	a. 129 ± 64.7 ng/g	↑	[47]
	Hashimoto thyroiditis(blood)	a. 16.5 ± 9.36 ng/g	a. 14.4 ± 7.82 ng/g		
	Hashimoto thyroiditis(urine)	a. 4.21 ± 3.66 ng/g	a. 3.69 ± 1.02 ng/g		
	Hypothyroid (serum)	a. 1.29 ± 0.27	a. 2.37 ± 0.34	↓	[35]
	Overweight (serum)	a. Pretreatment: 2.5 ± 0.4 6th month: 3.5 1.5	a. 2.1 ± 0.3	↑	[27]
	Obese (serum)	a. Pretreatment: 2.5 ± 0.3 6th month: 3.0 ± 1.4			
	Morbidly obese (serum)	a. Pretreatment: 2.7 ± 0.8 6th month: 3.1 ± 1.1			
**Cr**	Reference values: **serum**—<5.0 µg/L		Arup Laboratories		[20]
	Reference values: **blood**—18 years: <1.0 ng/mL		Mayo Clinic Laboratories		[21]
	Hypothyroid (serum)	a. 12.31 ± 1.229 μg/g	a. 8.422 ± 0.886 μg/g	↑	[31]
	Hypothyroid (serum)	b. 14.0 ±10.0 μg/L	b. 170.1 ±173.01 μg/L	↓	[44]
	Hypothyroid (serum)	a. 31.6 ± 11.3 μg/g	a. 18.9 ± 7.6 μg/g	↑	[39]
	Hypothyroid (serum)	a. 16.41 ± 3.65 μg/L	a. 0.42 ± 0.00 μg/L	↑	[43]
**Cd**	Reference values: -		Arup Laboratories		[20]
	Reference values: **blood**—<5.0 ng/mL		Mayo Clinic Laboratories		[21]
	Hypothyroid (serum)	a. 1.182 ± 0.114 μg/g	a. 0.423 ± 0.035 μg/g	↑	[31]
	Hypothyroid (serum)	b. 2.4 ± 3.1 μg/L	b. 45.3 ± 44.07 μg/L	-	[44]
	Hypothyroid (serum)	a. 1.1 ± 0.5 μg/g	a. 0.42 ± 0.31 μg/g	↑	[39]
	Hypothyroid (serum)	a. 0.50 ± 0.29 μg/L	a. 0.02 ± 0.00 μg/L	↑	[43]
	Hashimoto thyroiditis (thyroid tissue)	a. 50.7 ± 37.3 ng/g	a. 39.5 ± 29.7 ng/g	-	[47]
	Hashimoto thyroiditis(blood)	a. 0.25 ± 0.23 ng/g	a. 0.77 ± 0.56 ng/g	↓	
	Hashimoto thyroiditis(urine)	a. 0.78 ± 0.52 ng/g	a. 1.14 ± 1.12 ng/g	-	
**Pb**	Reference values: **blood (venous)**—≤4.9 μg/dL		Arup Laboratories		[20]
	Reference values: **blood (venous)**—<5.0 mcg/dL; **urine**—<2 mcg/24 h		Mayo Clinic Laboratories		[21]
	Hypothyroid (serum)	a. 12.84 ± 1.287 μg/g	a. 5.365 ± 0.761 μg/g	↑	[31]
	Hypothyroid (serum)	a. 5.58 ± 2.8 μg/g	a. 4.0 ± 2.5μg/g	↑	[39]
	Hypothyroid (whole blood)	a. Pretreatment: 3.44 ± 1.7 μg/dL Posttreatment: 2.74 ± 1.3 μg/dL	a. 4.18 ± 1.7 μg/dL	↑	[46]
	Hypothyroid (urine)	a. Pretreatment: 3.42 ± 2.1 μg/gCr Posttreatment: 6.25 ± 3.6 μg/gCr	a. 4.03 ± 2.2 μg/gCr		
	Hashimoto thyroiditis(thyroid tissue)	a. 88.2 ± 51.9 ng/g	a. 29.8 ± 23.0 ng/g	↑	[47]
	Hashimoto thyroiditis(blood)	a. 24.2 ± 12.0 ng/g	a. 12.3 ±7.65 ng/g		
	Hashimoto thyroiditis(urine)	a. 0.18 ± 0.13 ng/g	a. 0.53 ± 0.17 ng/g		
**Se**	Reference values: **serum**/**plasma**—23.0–190.0 μg/L; **urine**—12.0–40.0 µg/l		Arup Laboratories		[20]
	Reference values: **serum**—70–150 ng/mL; **blood**—≥150–241 ng/ml		Mayo Clinic Laboratories		[21]
	Autoimmune thyroiditis patients with subclinical hypothyroidism (serum)	a. Pretreatment: 67.7 ± 10.4 μg/dL Posttreatment: 66.2 ± 15.7 μg/dL	a. 83.7 ± 17.3 μg/dL	↓	[28]
	Hypothyroid (serum)	b. 49.3 ± 14.6 μg/L	b. 54.1 ± 11.13 μg/L	-	[44]
	Hashimoto disease(serum)	a. 0.75 ± 0.11 μM/L	a. 0.76 ± 0.12 μM/L	-	[38]
	Thyroid patients (serum)	a. 76.9 ± 18.8 μg/L	a. 85.1 ± 17.4 μg/L	↓	[34]
	Hashimoto thyroiditis(serum)	a. 0.72 ± 0.09 μM/L	5th and 95th centiles 0.7–1.2 μM/L	-	[45]
	Hypothyroidism (serum)	a. 98.6 ± 31.1 μg/L	a. 75.9 ± 11.07 μg/L	↑	[43]
	Hashimoto thyroiditis (serum)	median and interquartile ranges 51.9 (44.6–58.5) μg/L	median and interquartile ranges 56.0 (52.4–61.5) μg/;	-	[29]
	Hashimoto thyroiditis+LT4 (serum)	median and interquartile ranges 54.4 (44–63.4) μg/L	median and interquartile ranges 56.0 (52.4–61.5) μg/L	-	
	Hashimoto thyroiditis (serum)	a. 91.6 ± 17.7 μg/L	a. 97.2 ± 29.4 μg/L	-	[37]
	Hypothyroid (serum)	a. 85.9 ± 14.8 μg/L	a. 97.2 ± 29.4 μg/L	-	
	Hypothyroid(erythrocytes)	a. 0.50 ± 0.09 μg/g Dry Weight	a.0.47 ± 0.11 μg/g Dry Weight	-	[52]
	Hashimoto thyroiditis (thyroid tissue)	a. 102 ± 50.2 ng/g	a. 158 ± 102 ng/g	↓	[47]
	Hashimoto thyroiditis(blood)	a. 84.2 ± 28.9 ng/g	a. 95.4 ± 28.3 ng/g		
	Hashimoto thyroiditis(urine)	a. 65.1 ± 35.0 ng/g	a. 37.2 ± 15.5 ng/g		
	Hashimoto thyroiditis (serum)	a. 0.87 ± 0.29 μmol/L	a. 1.11 ± 0.37 μmol/L	↓	[40]
	Hashimoto thyroiditis (plasma)	a. 86.7 ± 37.1 μg/L	a. 89.3 ± 28.7 μg/L	-	[49]
	Autoimmune thyroid disease(serum)	a. 1st trimester: 66.6 ± 12.6 μg/L 2nd trimester: 59.0 ± 9.9 μg/L 3^rd^ trimester: 52.2 ± 11.6 μg/L	a. 1st trimester: 64.6 ± 14.6 μg/L 2nd trimester: 57.8 ± 14.0 μg/L 3rd trimester: 48.4 ± 11.3 μg/L	-	[24]
	Hypothyroid(venous blood)	a. 160.47 ± 25.76 µg/L	a. 206.72 ± 17.98 µg/L	-	[25]
	Hypothyroid (plasma)	a. 98.79 ± 13.63 μg/L	-		[48]
	Congenital hypothyroidism (serum)	a. 69.2 ± 17.4 μg/L	-		[26]
	Overweight (serum)	a. Pretreatment: 76.4 ± 21 6th month: 74.6 ± 30.2	a. 53.1 ± 27.9	↑	[27]
	Obese (serum)	a. Pretreatment: 78.7 ± 24.5 6th month: 60.5 ± 23.6			
	Morbidly obese (serum)	a. Pretreatment: 77.5 ± 25.7 6^th^ month: 67.2 ± 19.9			
	Pregnant women (serum)	a. 136.9 ± 47.9 μg/L	-		[30]
**F**	Reference values: **plasma**—<4.1 mcmol/L		Mayo Clinic Laboratories		[21]
	Iodine deficient adults (urine)	UF_SG_ 1.06 ± 1.11 mg/L	-		[51]
	Non-iodine deficient adults (urine)	UF_SG_ 0.91 ± 0.65 mg/L	-		
	Healthy children (serum)	mean I: 0.03 ppm II: 0.035 ppm III: 0.05 ppm	-		[41]
	Children with dental fluorosis (urine)	range 0.24–8.9 ppm	range (urine) 0.19–1.01 ppm range (serum) 0.02–0.09 ppm	↑	[42]
	Children with dental fluorosis (serum)	range 0.02–0. 77 ppm			
	Children without dental fluorosis (urine)	range 0.4–7.79 ppm			
	Children without dental fluorosis (serum)	range 0.03–0.75 ppm			
